# The Case for Junk DNA

**DOI:** 10.1371/journal.pgen.1004351

**Published:** 2014-05-08

**Authors:** Alexander F. Palazzo, T. Ryan Gregory

**Affiliations:** 1 University of Toronto, Department of Biochemistry, Toronto, Ontario, Canada; 2 University of Guelph, Department of Integrative Biology, Guelph, Ontario, Canada; University of Washington, United States of America

## Overview

With the advent of deep sequencing technologies and the ability to analyze whole genome sequences and transcriptomes, there has been a growing interest in exploring putative functions of the very large fraction of the genome that is commonly referred to as “junk DNA.” Whereas this is an issue of considerable importance in genome biology, there is an unfortunate tendency for researchers and science writers to proclaim the demise of junk DNA on a regular basis without properly addressing some of the fundamental issues that first led to the rise of the concept. In this review, we provide an overview of the major arguments that have been presented in support of the notion that a large portion of most eukaryotic genomes lacks an organism-level function. Some of these are based on observations or basic genetic principles that are decades old, whereas others stem from new knowledge regarding molecular processes such as transcription and gene regulation.

## Introduction

### The search for function in the genome

It has been known for several decades that only a small fraction of the human genome is made up of protein-coding sequences and that at least some noncoding DNA has important biological functions. In addition to coding exons, the genome contains sequences that are transcribed into functional RNA molecules (e.g., tRNA, rRNA, and snRNA), regulatory regions that control gene expression (e.g., promoters, silencers, and enhancers), origins of replication, and repeats that play structural roles at the chromosomal level (e.g., telomeres and centromeres).

New discoveries regarding potentially important sequences amongst the nonprotein-coding majority of the genome are becoming more prevalent. By far the best-known effort to identify functional regions in the human genome is the recently completed Encyclopaedia of DNA Elements (ENCODE) project [1], whose authors made the remarkable claim that a “biochemical function” could be assigned to 80% of the human genome [2]. Reports that ENCODE had refuted the existence of large amounts of junk DNA in the human genome received considerable media attention [3], [4]. Criticisms that these claims were based on an extremely loose definition of “function” soon followed [5]–[8] (for a discussion of the relevant function concepts, see [9]), and debate continues regarding the most appropriate interpretation of the ENCODE results. Nevertheless, the excitement and subsequent backlash served to illustrate the widespread interest among scientists and nonspecialists in determining how much of the human genome is functionally significant at the organism level.

### The origin of “junk DNA”

Although the term “junk DNA” was already in use as early as the 1960s [10]–[12], the term's origin is usually attributed to Susumu Ohno [13]. As Ohno pointed out, gene duplication can alleviate the constraint imposed by natural selection on changes to important gene regions by allowing one copy to maintain the original function as the other undergoes mutation. Rarely, these mutations will turn out to be beneficial, and a new gene may arise (“neofunctionalization”) [14]. Most of the time, however, one copy sustains a mutation that eliminates its ability to encode a functional protein, turning it into a pseudogene. These sequences are what Ohno initially referred to as “junk” [13], although the term was quickly extended to include many types of noncoding DNA [15]. Today, “junk DNA” is often used in the broad sense of referring to any DNA sequence that does not play a functional role in development, physiology, or some other organism-level capacity. This broader sense of the term is at the centre of most current debate about the quantity—or even the existence—of “junk DNA” in the genomes of humans and other organisms.

It has now become something of a cliché to begin both media stories and journal articles with the simplistic claim that most or all noncoding DNA was “long dismissed as useless junk.” The implication, of course, is that current research is revealing function in much of the supposed junk that was unwisely ignored as biologically uninteresting by past investigators. Yet, it is simply not true that potential functions for noncoding DNA were ignored until recently. In fact, various early commenters considered the notion that large swaths of the genome were nonfunctional to be “repugnant” [10], [16], and possible functions were discussed each time a new type of nonprotein-coding sequence was identified (including pseudogenes, transposable elements, satellite DNA, and introns; for a compilation of relevant literature, see [17]).

Importantly, the concept of junk DNA was not based on ignorance about genomes. On the contrary, the term reflected *known* details about genome size variability, the mechanism of gene duplication and mutational degradation, and population genetics theory. Moreover, each of these observations and theoretical considerations remains valid. In this review, we examine several lines of evidence—both empirical and conceptual—that support the notion that a substantial percentage of the DNA in many eukaryotic genomes lacks an organism-level function and that the junk DNA concept remains viable post-ENCODE.

## Genome Size and “The Onion Test”

**Figure 1:**
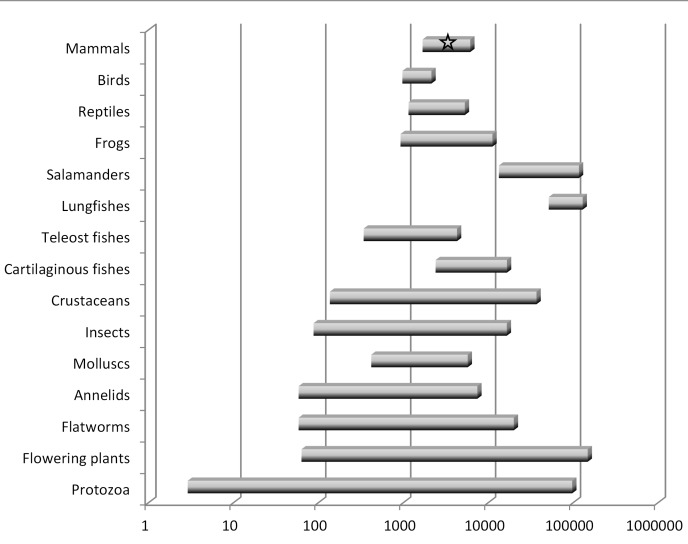
Summary of haploid nuclear DNA contents (“genome sizes”) for various groups of eukaryotes. This graph is based on data for about 10,000 species [18], [19]. There is a wide range in genome sizes even among developmentally similar species, and there is no correspondence between genome size and general organism complexity. Humans, which have an average-sized genome for a mammal, are indicated by a star. Note the logarithmic scale.

There are several key points to be understood regarding genome size diversity among eukaryotes and its relationship to the concept of junk DNA. First, genome size varies enormously among species [18], [19]: at least 7,000-fold among animals and 350-fold even within vertebrates. Second, genome size varies independently of intuitive notions of organism complexity or presumed number of protein-coding genes (Figure 1). For example, a human genome contains eight times more DNA than that of a pufferfish but is 40 times smaller than that of a lungfish. Third, organisms that have very large genomes are not few in number or outliers—for example, of the >200 salamander genomes analyzed thus far, all are between four and 35 times larger than the human genome [18]. Fourth, even closely related species with very similar biological properties and the same ploidy level can differ significantly in genome size.

These observations pose an important challenge to any claim that most eukaryotic DNA is functional at the organism level. This logic is perhaps best illustrated by invoking “the onion test” [20]. The domestic onion, *Allium cepa*, is a diploid plant (2n = 16) with a haploid genome size of roughly 16 billion base pairs (16 Gbp), or about five times larger than humans. Although any number of species with large genomes could be chosen for such a comparison, the onion test simply asks: if most eukaryotic DNA is functional at the organism level, be it for gene regulation, protection against mutations, maintenance of chromosome structure, or any other such role, then why does an onion require five times more of it than a human? Importantly, the comparison is not restricted to onions versus humans. It could as easily be between pufferfish and lungfish, which differ by ∼350-fold, or members of the genus *Allium*, which have more than a 4-fold range in genome size that is not the result of polyploidy [21].

In summary, the notion that the majority of eukaryotic noncoding DNA is functional is very difficult to reconcile with the massive diversity in genome size observed among species, including among some closely related taxa. The onion test is merely a restatement of this issue, which has been well known to genome biologists for many decades [18].

## Genome Composition

Another important consideration is the composition of eukaryotic genomes. Far from being composed of mysterious “dark matter,” the characteristics of the sequences constituting 98% or so of the human genome that is nonprotein-coding are generally well understood.

### Transposable elements

By far the dominant type of nongenic DNA are transposable elements (TEs), including various well-described retroelements such as Short and Long Interspersed Nuclear Elements (SINEs and LINEs), endogenous retroviruses, and cut-and-paste DNA transposons. Because of their capacity to increase in copy number, transposable elements have long been described as “parasitic” or “selfish” [22], [23]. However, the vast majority of these elements are inactive in humans, due to a very large fraction being highly degraded by mutation. Due to this degeneracy, estimates of the proportion of the human genome occupied by TEs has varied widely, between one-half and two-thirds [24], [25]. Larger genomes, such as those of salamanders and lungfishes, almost certainly contain an even more enormous quantity of transposable element DNA [26], [27].

Many examples have been found in which TEs have taken on regulatory or other functional roles in the genome [28]. In recognition of the more complex interactions between transposable elements and their hosts, Kidwell and Lisch proposed an expansion of the “parasitism” framework where each TE can be classified along a spectrum from parasitism to mutualism [29]. Nevertheless, there is evidence of organism-level function for only a tiny minority of TE sequences. It is therefore not obvious that functional explanations can be extrapolated from a small number of specific examples to all TEs within the genome.

### Highly repetitive DNA

Another large fraction of the genome consists of highly repetitive DNA. These regions are extremely variable even amongst individuals of the same population (hence their use as “DNA fingerprints”) and can expand or contract through processes such as unequal crossing over or replication slippage. Many repeats are thought to be derived from truncated TEs, but others consist of tandem arrays of di- and trinucleotides [30]. As with TEs, some highly repetitive sequences play a role in gene regulation (for example, [31]). Others, such as telomeric- and centromeric-associated repeats [32], [33], play critical roles in chromosomal maintenance. Despite this, there is currently no evidence that the majority of highly repetitive elements are functional.

### Introns

According to Gencode v17, about 40% of the human genome is comprised of intronic regions; however, this figure is likely an overestimate as it includes all annotated events. It is also important to note that a large fraction of TEs and repetitive elements are found in introns. Although introns can increase the diversity of protein products by modulating alternative splicing, it is also clear that the vast majority of intronic sequence evolves in an unconstrained way, accumulating mutations at about the same rate as neutral regions. Although the median intron size in humans is ∼1.5 kb [30], data suggest that most of the constrained sequence is confined to the first and last 150 nucleotides [34].

### Pseudogenes

The human genome is also home to a large number of pseudogenes. Estimates of the total number range from 12,600 to 19,700 [35]. These include both “classical” pseudogenes (direct duplicates, of the sort imagined by Ohno [13]) and “processed” pseudogenes, which are reverse transcribed from mRNA [36]. Once again, although some pseudogenes have been co-opted for organism-level function (for example see [37]), most are simply evolving without selective constraints on their sequences and likely have no function [38].

### Conserved sequences

Several analyses of sequence conservation between humans and other mammals have found that about 5% of the genome is conserved [1], [39]–[42]. It is possible that an additional 4% of the human genome is under lineage-specific selection pressure [39]; however, this estimate appears to be somewhat questionable [43], [44] (also see [45]). Ignoring these problems, the idea that 9% of the human genome shows signs of functionality is actually consistent with the results of ENCODE and other large-scale genome analyses.

Besides protein-coding sequences (including associated untranslated regions), which make up 1.5%–2.5% of the human genome [24], data from ENCODE suggest that conserved long noncoding RNAs (lncRNAs) are generated from about 9,000 loci that add up to less than an additional 0.4% [46], [47]. Thus, even if a vast new untapped world of functional noncoding RNA is discovered, this will probably be transcribed from a small fraction of the human genome.

At first blush, sequences that are bound by transcription factors (TFs) appear to be very abundant, making up about 8.5% of the genome according to ENCODE [2]. This number, however, is an estimate of regions that are hypersensitive to DNase I treatment due to the displacement of nucleosomes by TFs. As pointed out by others [6], these regions are annotated as being several hundreds of nucleotides long and are thus much larger than the actual size of individual TF-binding motifs, which are typically 10 bp in length [48]. By ENCODE's own estimates, less than half of the nucleotide bases in these DNase I hypersensitivity regions contain actual TF recognition motifs [2], and only 60% of these are under purifying selection [49]. Others have found that weak and transient TF-binding events are routinely identified by chromatin IP experiments despite the fact that they do not significantly contribute to gene expression [50]–[53] and are poorly conserved [53]. Given that experiments performed in a diverse number of eukaryotic systems have found only a small correlation between TF-binding events and mRNA expression [54], [51], it appears that in most cases only a fraction of TF-binding sites significantly impacts local gene expression.

In summary, most of the major constituents of the genome have been well characterized. The majority of human DNA consists of repetitive, mutationally degraded sequences. There are unambiguous examples of nonprotein-coding sequences of various types having been co-opted for organism-level functions in gene regulation, chromosome structure, and other roles, but at present evidence from the published literature suggests that these represent a small minority of the human genome.

## Evolutionary Forces

To understand the current state of the human genome, we need to examine how it evolved, and as Michael Lynch once wrote, “Nothing in evolution makes sense except in the light of population genetics” [55]. Unfortunately, concepts that have been generated by this field have not been widely recognized in other domains of the life sciences. In particular, what is underappreciated by many nonevolution specialists is that much of molecular evolution in eukaryotes is primarily the result of genetic drift, or the fixation of neutral mutations. This view has been widely appreciated by molecular evolutionary biologists for the past 35 years.

### The nearly neutral theory of molecular evolution

An important development in the understanding of how various evolutionary forces shape eukaryotic genes and genomes came with the theories developed by Kimura, Ohta, King, and Jukes. They demonstrated that alleles that were slightly beneficial or deleterious behaved like neutral alleles, provided that the absolute value of their selection coefficient was smaller than the inverse of the “effective” population size [56]–[59]. In other words, it is important to keep in mind population size when thinking about whether deleterious mutations are subjected to purifying selection.

It is also important to realize that the “effective” population size is dependent on many factors and is typically much lower than the total number of individuals in a species [55]. For humans it has been estimated that the historical effective population size is approximately 10,000, and this is on the low side in comparison to most metazoans [60]. Given the overall low figures for multicellular organisms in general, we would expect that natural selection would be powerless to stop the accumulation of certain genomic alterations over the entirety of metazoan evolution. One type of mutation that fits this description is intergenic insertions, be they transposable elements, pseudogenes, or random sequence [55]. The creation and loss of TF-binding motifs or cryptic transcriptional start sites in these same intergenic regions will equally be invisible to natural selection, provided that these do not drastically alter the expression of any nearby genes or cause the production of stable toxic transcripts. Thus, a central tenet of the nearly neutral theory of molecular evolution is that extraneous DNA sequences can be present within genomes, provided that they do not significantly impact the fitness of the organism.

### Genetic load

It has long been appreciated that there is a limit to the number of deleterious mutations that an organism can sustain per generation [61], [62]. The presence of these mutations is usually not harmful, because diploid organisms generally require only one functional copy of any given gene. However, if the rate at which these mutations are generated is higher than the rate at which natural selection can weed them out, then the collective genomes of the organisms in the species will suffer a meltdown as the total number of deleterious alleles increases with each generation [63]. This rate is approximately one deleterious mutation per generation. In this context it becomes clear that the overall mutation rate would place an upper limit to the amount of functional DNA. Currently, the rate of mutation in humans is estimated to be anywhere from 70–150 mutations per generation [64], [65]. By this line of reasoning, we would estimate that, at most, only 1% of the nucleotides in the genome are essential for viability in a strict sequence-specific way. However, more recent computational models have demonstrated that genomes could sustain multiple slightly deleterious mutations per generation [66]. Using statistical methods, it has been estimated that humans sustain 2.1–10 deleterious mutations per generation [66]–[68]. These data would suggest that at most 10% of the human genome exhibits detectable organism-level function and conversely that at least 90% of the genome consists of junk DNA. These figures agree with measurements of genome conservation (∼9%, see above) and are incompatible with the view that 80% of the genome is functional in the sense implied by ENCODE. It remains possible that large amounts of noncoding DNA play structural or other roles independent of nucleotide sequence, but it far from obvious how this would be reconciled with “the onion test.”

### The evolution of the nucleus

When dealing with the evolution of any lineage, one must also keep in mind unique events, also known as historical contingencies, which constrain and shape subsequent evolutionary trajectories [69]. One of these key events in our own ancestry was the evolution of the eukaryotic nucleus. A further examination of why the nucleus evolved and how this altered cellular function may generate significant insights into the current shape of the eukaryotic genome.

One important event in early eukaryotic evolution was the development of a symbiotic relationship between the α-proteobacteria progenitor of mitochondria and an archaebacteria-like host [70], [71]. As with most endosymbiotically derived organelles [72], DNA was transferred from mitochondria to the host. In this way, Group II introns, which are still found in both mitochondria and α-proteobacteria [73], invaded the host genome. Group II introns are parasitic DNA fragments that replicate when they are transcribed, typically as part of a larger transcript. The intron then folds into a catalytic ribozyme that splices itself out of the precursor transcript and then reinserts itself at a new genomic locus by reversing the splicing reaction. Importantly, functional fragments of Group II introns can splice out inactive versions in a trans-splicing reaction [74], [75]. As described elsewhere, it is likely that Group II introns proliferated and evolved into two populations: inactivated copies that could be nonetheless spliced out in trans, and active fragments that promoted splicing of the former group. This latter group eventually evolved into the spliceosomal snRNAs [75]–[77]. This idea is supported by not only structural, catalytic, and functional similarities between Group II introns and snRNAs [78], [79] but also by the fact that expression of the U5 snRNA rescues the splicing of Group II introns that lack the corresponding U5-like region [80].

It is likely that the proliferation of trans-splicing triggered the spatial segregation of RNA processing (the nucleoplasm) from the translation machinery (the cytoplasm) [77]. This subdivision ensured that mRNAs were properly spliced before they encountered the translation machinery. Not only would this segregation prevent translating ribosomes from interfering with the splicing reaction (and vice versa) but would also prevent the translation of incompletely processed mRNAs, which often encode toxic proteins [81], [82]. Importantly, the segregation of translation from both transcription and RNA processing provided an opportunity for nuclear quality-control processes to eliminate misprocessed and spurious transcripts that did not meet the minimal requirements of “mRNA identity” (see below) before these RNAs ever encountered a ribosome. This in turn permitted intergenic DNA and cryptic transcriptional start sites to proliferate with minimal cost to the fitness of the organism. It should also be noted that the increase in ATP regeneration due to mitochondrial-derived metabolic pathways provided the surplus energy that is required to support an expansion not only in genome size and membranes [83], [84] but also wasteful transcription. Thus, by several independent mechanisms, the acquisition of mitochondria likely allowed the expansion of nonfunctional intergenic DNA and the evolution of a noisy transcriptional system.

## Gene Expression in Eukaryotes

### Eukaryotic transcription is inherently noisy

**Figure 2:**
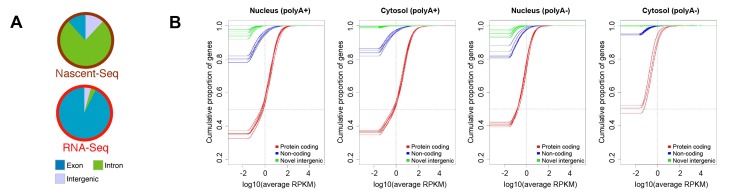
Levels of protein-coding and intergenic RNAs in mammalian cells. (A) Analysis of nascent and total poly(A)+ RNA levels from mouse liver nuclei. Nascent (i.e., polymerase-associated) RNA and poly(A)+ RNA were isolated from mouse liver nuclei and analyzed by high-throughput sequencing. Individual reads were categorized by their source. Exonic and intronic are from known referenced genes (i.e., “RefSeq” genes), while intergenic originate from nonreferenced loci (i.e., “non-RefSeq”) in the mouse genome. Reproduced from [85]. (B) Empirical Cumulative Distribution Function (ECDF) of transcript expression in each cell compartment as determined by the ENCODE consortia. Results for RNA that either contain (“polyA+”) or lack (“polyA−”) a poly(A)-tail in the nucleus and cytosolic fractions are shown. Each human cell line that was analyzed is represented by three lines, one for each pool of RNA (red for protein-coding RNAs, blue for lncRNAs [“noncoding”], and green for intergenic transcripts [“novel intergenic”]). The lines indicate the cumulative fraction of RNAs in a given pool (y-axis) that are expressed at levels that are equal or less than the reads per kilobase per million mapped reads (RPKM) on the x-axis. Total numbers in each pool are as follows: reference protein coding genes: 20,679, loci producing lncRNAs: 9,277, and regions producing intergenic transcripts: 41,204. Transcripts with expression levels of 0 RPKM were adjusted to an artificial value of 10^−6^ RPKM so that the onset of each graph represents the fraction of nonexpressed genes or loci. Note that 1–4 RPKM is approximately equivalent to one copy per tissue culture cell [46], [129]. Using this figure, one can easily deduce that the vast majority of intergenic transcripts are present at levels less than one copy per cell. Reproduced with permission from [46].

One of the most widely discussed discoveries of the past decade of transcriptome analysis is that much of the metazoan genome is transcribed at some level (although this, too, was already recognized in rough outline in the 1970s [15]). When nascent transcripts from mouse have been analyzed by deep sequencing, the total number of reads that map to intergenic loci is almost equivalent to the number mapping to exonic regions (Figure 2A, reproduced from reference [85]). This is consistent with the observation that a large fraction of the cellular pool of RNA Polymerase II is associated with intergenic regions [86] and that transcription can be initiated at random sequences (see Figure S4 in [87]) and nucleosome-free regions [88], [89]. Strikingly, when one examines the steady state level of polyadenylated RNA, very little maps to intergenic regions (Figure 2A, 2B, the latter reproduced from reference [46]; also see [85], [90]–[92]). In fact, when one eliminates the ∼9,000 transcript species that are thought to be derived from conserved lncRNA, then most of the annotated noncoding polyadenylated RNAs are present at levels below one copy per cell and are found exclusively in the nucleus (Figure 2B). The situation is no better in the unpolyadenylated pool, in which the amount of lncRNA and intergenic RNA is practically insignificant, especially in the cytoplasmic pool (Figure 2B). In aggregate, these data indicate that the majority of intergenic RNAs are degraded almost immediately after transcription. Consistent with this idea, the level of intergenic transcripts increase when RNA degradation machinery is inhibited [93]–[101]. Although pervasive transcription has been used as an argument against junk DNA [3], [4], it is in fact entirely in line with the idea that intergenic regions are evolving under little-to-no constraint, especially when one considers that this intergenic transcription is unstable.

### Identifying mRNA from intergenic transcription

A common theme that has emerged from the study of mRNA synthesis is that various steps in RNA synthesis and processing are biochemically coupled. In other words, cellular machineries that participate in one biochemical activity also promote subsequent steps. For example, during the splicing of the 5′most intron, the spliceosome collaborates with the 5′cap binding complex to deposit nuclear export factors onto the 5′end of the processed transcript [102], [103], and this helps to explain why splicing enhances the nuclear export of mRNA [104]–[106]. Countless other examples of coupling exist (for reviews, see [107]–[111]).

The ultimate goal of these coupling reactions is to sort protein-coding RNAs (i.e. mRNA) from intergenic transcripts [111], [112]. Given that, on average, protein-coding genes have eight introns [30], while the majority of annotated ENCODE intergenic transcripts tend not to be spliced [46], introns help distinguish these two populations and thus serve as “mRNA identity” markers. These mRNA identity features activate coupling reactions, which in turn promote the further processing, nuclear export, and translation of a particular transcript. Likewise, other classes of functional RNAs (e.g., tRNAs and snRNAs) have their own identity elements [113]. In contrast, transcripts that lack identity elements are targeted for degradation. In agreement with this model, intronless RNA molecules that have a random sequence are poorly exported from the nucleus and have a very short half-life [114], [115]. In contrast, intronless mRNAs have specialized motifs that promote their nuclear export [105], [116]–[119].

In light of the fact that many functional lncRNAs serve a role in regulating chromatin structure or transcription, it is not surprising that most localise to the nucleoplasm [46]. One would predict that lncRNAs contain a differential set of identity elements that not only serve to prevent their decay but also retain them in the nucleus. This would especially be critical for lncRNAs that are spliced. Despite this, the elements that regulate the localization and stability of these RNAs have received little attention, but can be informed by the view that they may have their own identity markers.

It is also important to point out that eukaryotes have other mechanisms that either degrade aberrant mRNAs (e.g., nonsense-mediated decay) or limit the amount of intergenic transcription (e.g., heterochromatin). Nevertheless, eukaryotes appear to have evolved an intricate network of coupling reactions that are required to cope with a large burden of junk RNA. These findings are consistent with the idea that eukaryotic genomes are filled with junk DNA that is transcribed at a low level.

### An alternative view of transcription and conservation?

In an attempt to counter the argument that sequence conservation is a prerequisite for functionality, it has been recently proposed that certain transcriptional events may serve some role in regulating cellular function, despite the fact that the sequence of the transcriptional product is unconstrained [120]. Indeed, this view is in line with the findings that the transcription of certain yeast genes is inhibited as a consequence of the production of cryptic unstable transcripts originating from upstream and/or downstream promoters (for a review see [121]). Other examples have linked the generation of cryptic unstable transcripts to chromatin modifications [101], [122], DNA methylation [123], and DNA stability [124]. However, it remains unclear whether the majority of unstable noncoding RNAs have any effect on DNA or chromatin, let alone contribute to the fitness of the organism. In the cases where cryptic unstable transcriptional events impact gene expression, they usually consist of short transcripts that are synthesized from regions around the transcriptional start sites or within the gene itself [121]. Indeed most of the available data are consistent with the fact that transcriptional start sites are promiscuous, often generating bidirectional transcription [100], [101], and that subsequent coupling processes, such as the interaction between promoter-associated complexes and 3′end processing factors, are required to enforce proper transcriptional directionality [125]. Other unstable transcripts function to promote or maintain heterochromatin formation in the vicinity of the transcriptional site, likely because these regions produce toxic transcripts [122]. Although this form of transcription has a function (viz., to maintain a repressive state), it is not clear that the elimination of these regions would have any effect on the organism [8]. The transcription of other short unstable transcripts, mostly produced from enhancer regions, has been shown to promote gene expression [126]; however, again these “enhancer RNAs” are transcribed from a small fraction of the total genome [127]. As stated by others [128], it is imperative that those who claim that the vast majority of intergenic transcription is functional test their hypotheses. In the absence of this evidence, the declaration that we are in the midst of a paradigm shift with regards to eukaryotic genomes and gene expression [120] seems premature.

## Concluding Remarks

For decades, there has been considerable interest in determining what role, if any, the majority of the DNA in eukaryotic genomes plays in organismal development and physiology. The ENCODE data are only the most recent contribution to a long-standing research program that has sought to address this issue. However, evidence casting doubt that most of the human genome possesses a functional role has existed for some time. This is not to say that none of the nonprotein-coding majority of the genome is functional—examples of functional noncoding sequences have been known for more than half a century, and even the earliest proponents of “junk DNA” and “selfish DNA” predicted that further examples would be found. Nevertheless, they also pointed out that evolutionary considerations, information regarding genome size diversity, and knowledge about the origins and features of genomic components do not support the notion that all of the DNA must have a function by virtue of its mere existence. Nothing in the recent research or commentary on the subject has challenged these observations.
